# Implementation competence for evidence-based practice among vascular access specialist team nurses: a mixed-method study

**DOI:** 10.1186/s12912-026-04783-w

**Published:** 2026-06-17

**Authors:** Ismael Fernández-Fernández, Ana Casaux-Huertas, Enrique Castro-Sánchez, Antonio Zamudio-Sánchez, Ian Blanco-Mavillard

**Affiliations:** 1 Manacor Hospital, Manacor, Balearic Islands 07500 Spain; 2 Department of Nursing, University of Valladolid, Campus María Zambrano, Segovia, 40005 Spain; 3 Health Protection Research Unit in Healthcare-Associated Infection and Antimicrobial Resistance, Imperial College London, South Kensington Campus, London, SW7 2AZ UK; 4 National Infection Prevention and Control Team, UK Health Security Agency, 10 South Colonnade, London E14 4PU London, UK; 5 Hospital Regional Universitario de Málaga, Málaga, Andalusia 29009 Spain; 6 IBIMA Plataforma Bionand, Research Group BE-23, Málaga, Andalusia 29590 Spain; 7 IBIMA Plataforma Bionand, Research Group C-13, Málaga, Andalusia 29590 Spain

**Keywords:** Evidence-based practice, Implementation competence, Vascular access, Nurse competence, Professional capability

## Abstract

**Background:**

Evidence-based practice (EBP) is central to high-quality nursing care and patient safety, yet its effective adoption and sustainability depend on nurses’ capability to translate evidence into routine practice. Vascular access specialist teams (VASTs) play a key role in implementing evidence-based interventions to reduce complications associated with intravenous therapy, but little is known about their capability to enact and sustain EBP in complex organisational contexts. This study aimed to assess implementation competencies for EBP among VAST nurses in Spain.

**Methods:**

A two-stage, mixed-methods study was conducted in 2023. The quantitative stage involved a national cross-sectional survey of VAST nurses using an adapted questionnaire informed by behavioural frameworks relevant to EBP adoption. The qualitative stage consisted of in-depth semi-structured interviews with a purposive sample of participants. Quantitative data were analysed using descriptive and multivariate methods, and qualitative data through thematic analysis and integrated using triangulation.

**Results:**

Fifty-one VAST nurses completed the questionnaire, reporting high self-perceived competence in domains such as social/professional role and identity (mean 4.7/5), knowledge (4.3), and collective context (4.2) for EBP. Lower scores were observed in contextual and organisational domains (mean 3.9), reflecting structural barriers to sustained EBP. Knowledge correlated positively with beliefs about capabilities (ρ = 0.574), and behavioural regulation (ρ = 0.591). Qualitative findings revealed five themes: expanded professional roles, experiential learning of implementation skills, context-sensitive strategies, organisational barriers, and enabling conditions. Participants described intuitive engagement with EBP despite lacking formal training, highlighting a gap between individual motivation and organisational support.

**Conclusions:**

VAST nurses in Spain show strong intrinsic motivation and emerging capability to lead and support EBP, but limited formal training and weak organisational infrastructure restrict their ability to enact and sustain implementation activities. Strengthening nurses’ EBP capability through structured implementation training, alongside organisational strategies that formalise and support the VAST role, may improve the adoption and sustainability of evidence-based vascular access care.

**Supplementary Information:**

The online version contains supplementary material available at https://doi.org/10.1186/s12912-026-04783-w.

**Table:** 

Contributions to literature
• This study provides the first empirical characterisation of implementation competence for evidence-based practice (EBP) among vascular access specialist teams (VASTs), a workforce essential to evidence-based vascular access care but previously unexplored in relation to EBP capability.
• By integrating quantitative and qualitative evidence, the study identifies key behavioural and contextual mechanisms, such as self-efficacy, behavioural regulation, and organisational support, that influence nurses’ capability to implement and sustain EBP, offering insights into how EBP capability develops within specialised nursing teams.
• Findings reveal a gap between strong individual motivation for EBP and limited organisational capacity to support it, underscoring the need for structured education in implementation and EBP, as well as system-level arrangements that formally embed VAST roles within quality and safety strategies.
• By foregrounding the experiences and challenges of vascular access specialist nurses, this study provides actionable insights for nursing leadership, workforce development, and policy initiatives, highlighting the need to align individual motivation for EBP with organisational capacity to support its sustained implementation.

## Background

Catheters are intravascular devices commonly used in healthcare to administer intravenous therapy and represent one of the most frequently used medical devices in hospitals [1]. Nearly three quarters of hospitalised patients require at least one vascular access device (VAD) during their stay [1], making vascular access care a critical component of nursing practice with direct implications for patient safety [2]. The use of VADs can lead to complications such as infections, mechanical obstructions, thrombosis, phlebitis, infiltration, and extravasation [3–5]. Catheter-related bloodstream infections (CRBSI) remain one of the most serious healthcare-associated complications worldwide, with reported prevalence ranging from 0.4 to 1.2% depending on device type and clinical context and are associated with high morbidity, mortality and healthcare costs [6–8].

The selection, insertion, management, and maintenance of VADs constitute a complex, multifaceted process that requires ongoing clinical judgement and coordination by nurses and other healthcare professionals [9]. Some of the factors influencing VAD outcomes include patient diagnosis, vascular condition, characteristics and duration of the therapy administered, VAD availability, healthcare professional competence, patient preferences, organisational culture, and established standards [10, 11]. This complexity contributes to variability in practice and poses a significant challenge for the consistent application of evidence-based practice (EBP) in vascular access care [2, 12–14]. To address variability and improve patient outcomes, healthcare organisations have increasingly promoted the development and institutionalisation of vascular access specialist teams (VASTs) [15–17]. These teams typically involve experienced nurses with advanced knowledge and skills, who comprehensively assess patients to optimise VAD selection, insertion, maintenance, and removal, while adapting care to patients’ evolving clinical needs [11]. Beyond technical proficiency, VAST nurses frequently act as advisors and consultants to other professionals [18], supporting decision-making and recommending evidence-based interventions to address gaps between best evidence and routine practice [19, 20]. As such, VASTs play a central role in facilitating the uptake of evidence-based vascular access care within multidisciplinary teams [19]. However, despite their recognised clinical value, the implementation and formal integration of VASTs within hospital organisations remain inconsistent, with reported challenges related to role definition, institutional support, and sustainability across different healthcare settings [21, 22].

VASTs provide substantial benefits to patients by ensuring safe and effective vascular access management. Beyond direct patient care, these teams offer expert support to multidisciplinary healthcare teams that rely on their specialised knowledge [21]. In addition, VASTs contribute to improving healthcare delivery processes by identifying and optimising clinical pathways related to vascular access and intravenous therapy. To achieve these improvements, VAST nurses must mobilise the best available evidence and translate it into clinical practice. This requires not only clinical expertise, but also competence in implementing and sustaining EBP and practice change, enabling them to effectively bridge the gap between evidence and routine care [23]. Implementation competence for EBP can be understood as the ability to purposefully apply evidence-informed strategies and behavioural skills to promote practice change within specific organisational contexts. According to the implementation framework, this competence aligns with the facilitator role – integrating evidence, contextual knowledge, stakeholder skills and motivations, and facilitation strategies to enable change and sustainability [24].

Despite these expectations, there is limited empirical knowledge about how VAST nurses develop, enact, and perceive these implementation-related capacities. Theoretical models such as the COM-B or the theoretical domains framework offer a behavioural lens to understand the determinants of implementation competence, characterising it beyond technical or clinical skill [23]. As a specialised core component of intravenous care, VASTs, along with the rest of the healthcare team, face challenges regarding the dissemination, adoption, and sustainability of evidence, resource adequacy, and care management decisions [25–27]. Although implementation science has emerged as a valuable field to support healthcare professionals in addressing these challenges and to perform their roles effectively [28–30], most research in vascular access has focused on clinical outcomes rather than on the professional and organisational capacities required to translate evidence into routine nursing practice [19, 21–23]. Addressing this gap is essential to strengthen evidence-based vascular access care and improve patient safety. By examining implementation competence for EBP among VAST nurses, this study aims to inform educational, organisational, and policy strategies that support nurses in leading and sustaining EBP. Accordingly, this study assesses implementation competence for EBP among VAST nurses in Spain, exploring their experiences, attitudes, and perceptions of organisational support, with a particular focus on their facilitative role in promoting practice change within healthcare organisations.

## Methods

This mixed-methods study was conducted in two stages. The quantitative stage involved adaptation of the questionnaire developed by Huijg et al. [31] to the Spanish context. The qualitative stage used semi-structured in-depth interviews to explore the role of VAST nurses in facilitating change and implementing quality improvement interventions. The rationale for this multiple methodology is the complexity of implementation science itself, its intrinsic characteristics within this organisational context, skills acquisition by healthcare professionals, and the way these skills are developed in clinical practice through a facilitative role within VASTs. Each stage of the research is described in detail below:

## Quantitative stage

### Study design

We conducted a multicentre, descriptive, cross-sectional study, using an online questionnaire based on Huijg et al. [31]. We adapted the original questionnaire to evaluate the implementation skills of quality improvement interventions among VAST nurses in the Spanish context. As a similar study has never been done before in our context, we conducted this exploratory study, and the domains selected are those that we considered to fit our practice environment on a clinical, social, and cultural level.

### Participants

Eligibility required employment as part of a VAST nursing team for at least three months at the time of the study, in any healthcare organisation in Spain, whether private or state funded. Participation in the study was voluntary and did not involve any financial compensation.

### Participant recruitment

We employed a recruitment matrix to guide initial contact with VASTs across Spain, structured by autonomous community, province capital, and major city, prioritising leading hospitals within each geographical unit. Hospitals were contacted sequentially; when no response was obtained, no VAST was available, or participation was declined, alternative centres were approached.

In practice, recruitment was pragmatic and dependent on institutional response and accessibility. Additionally, participants were recruited through dissemination of the study via social networks (Twitter/X).

Once initial contact was established, VAST nurses were provided with detailed information about the study via email. Those who agreed to participate received a link to the online questionnaire for completion, which also allowed them to express their interest in participating in the qualitative stage of the study.

### Procedure / data collection

We collected data using an online questionnaire distributed to participating VAST nurses during March – June 2023 through the REDCap platform (Research Electronic Data Capture). All participating institutions and VASTs were contacted prior to data collection to inform them about the study and obtain informed consent.

### Measuring instrument and variables

The original questionnaire [31], grounded in the Theoretical Domains Framework (TDF), was adapted to focus on implementation skills in improvement interventions and included five sociodemographic and organisational items (age, gender, years of experience as a nurse, years of experience as a VAST nurse, and hospital/facility of work), as well as 29 items concerning implementation science distributed across seven domains (D1. Knowledge; D2. Skills; D3. Social/professional role and identity; D4. Beliefs about capabilities; D5. Context; D6. Collective context; D7. Behavioural regulation). The seven domains were scored on a 5-point Likert scale, ranging from 1 (low relevance) to 5 (high relevance) (see Appendix 1 for domain definitions).

The questionnaire was translated and adapted to the Spanish context with attention to conceptual clarity and relevance to vascular access practice. Given the exploratory nature of the study and the absence of validated instruments specifically designed to assess implementation competence in this context and language, a formal transcultural validation process was not undertaken.

### Sampling strategy

The sample was obtained using a purposive sampling approach with nurses working in VASTs at a national level. A recruitment matrix was designed to maximise geographical representation across the 17 autonomous communities and the 2 autonomous cities of Spain, prioritising leading hospitals within each region, including those located in provincial capitals. However, recruitment was conducted sequentially and pragmatically, depending on institutional response, the availability of VAST teams, and willingness to participate.

### Statistical analysis

Descriptive statistics were used, with categorical variables expressed as frequencies and percentages, and continuous variables as means, medians, standard deviations (SDs), and interquartile ranges (IQRs). Bivariate analyses were conducted using parametric or non-parametric tests, depending on data distribution, including correlation tests, ANOVA, and chi-squared tests. Missing data were not included in the analysis. The statistical significance threshold was set at *p* < 0.05. All statistical analyses were conducted using SPSS software (version 25; IBM Corp., Chicago Illinois, USA).

## Qualitative stage

### Study design

We conducted a qualitative study, using a semi-structured interview to explore perceptions, experiences, and knowledge of implementation science among VAST nurses, identifying the facilitator role of these professionals.

### Participant selection and recruitment

As detailed above, VAST nurses who voluntarily provided their contact details (email address or telephone number) and consented to participate in the qualitative stage were eligible. These participants were considered as key informants to help identify and recruit other suitable participants, using a snowball approach. We highlighted characteristics of sought after participants, for example, background and experience to enhance the potential richness of the interviews. Participation in the study was voluntary and did not involve any financial compensation for any of the participants. Participant selection, recruitment, and interviews were finalised once thematic saturation was reached.

### Data collection

All semi-structured interviews were conducted by two researchers (IB-M and EC-S, two members of the research team) using an interview guide (Appendix 2). The semi-structured interviews were conducted online using MS Teams during the second half of 2023 and first of 2024, at times convenient to the participants and lasted approximately 45 min. The interviews were recorded and automatically transcribed verbatim by the online tool. The researchers reviewed the transcriptions against the recordings and returned them to participants for fidelity and clarification. The transcriptions were then anonymised prior to analysis. The researchers also took contemporary notes during and after the interview.

### Analysis

We conducted a thematic analysis, following a continuous iterative familiarisation process [32]. In the inductive phase, we examined the transcripts for units of meaning that alluded to how the VAST facilitation process unfolded on the hospital wards and then coded them. These codes were grouped into broader categories and subcategories. Three researchers independently coded each transcript of the semi-structured interviews. During the deductive phase, the data were analysed according to the aspects proposed in the theoretical framework and the literature review. A first coding framework was applied to the interviews, which was once again returned to respondents for codes and prominent theme validation.

We employed several triangulation strategies combined to compare the results obtained and build a code tree, triangulated by three researchers (AC-H, IB-M, and EC-S), to further enhance the methodological rigour of our analysis [33]. Regarding the methods, we compared the information from the interviews, the codes applied by the researchers, and the participants’ reviews to identify coherence and discrepancies in the narrative (member checking). Regarding the data, two members of the research team with ample experience in implementation science (IB-M and EC-S) shared and discussed their findings. Another strategy to enhance rigour was to ensure meticulous adherence to procedure and that the researchers’ reflections on methodological decisions made throughout the study are properly recorded, taking their dual status as clinicians and co-researchers into consideration [34].

### Research and reflexivity team

The research team’s knowledge of implementation processes informed the interpretation and contextualisation of the analysis. Two researchers (IB-M and EC-S) with previous experience in evidence implementation, social research, and vascular access coordinated and conducted the semi-structured interviews, which facilitated the establishment of a relationship with participants and encouraged open and frank discussion. IF-F is a doctoral student in the *Investigación Traslacional en Salud Pública y Enfermedades de Alta Prevalencia* (Translational Research in Public Health and High Prevalence Diseases) doctoral programme at the University of the Balearic Islands.

## Results

### Quantitative stage

**Fig. 1:**
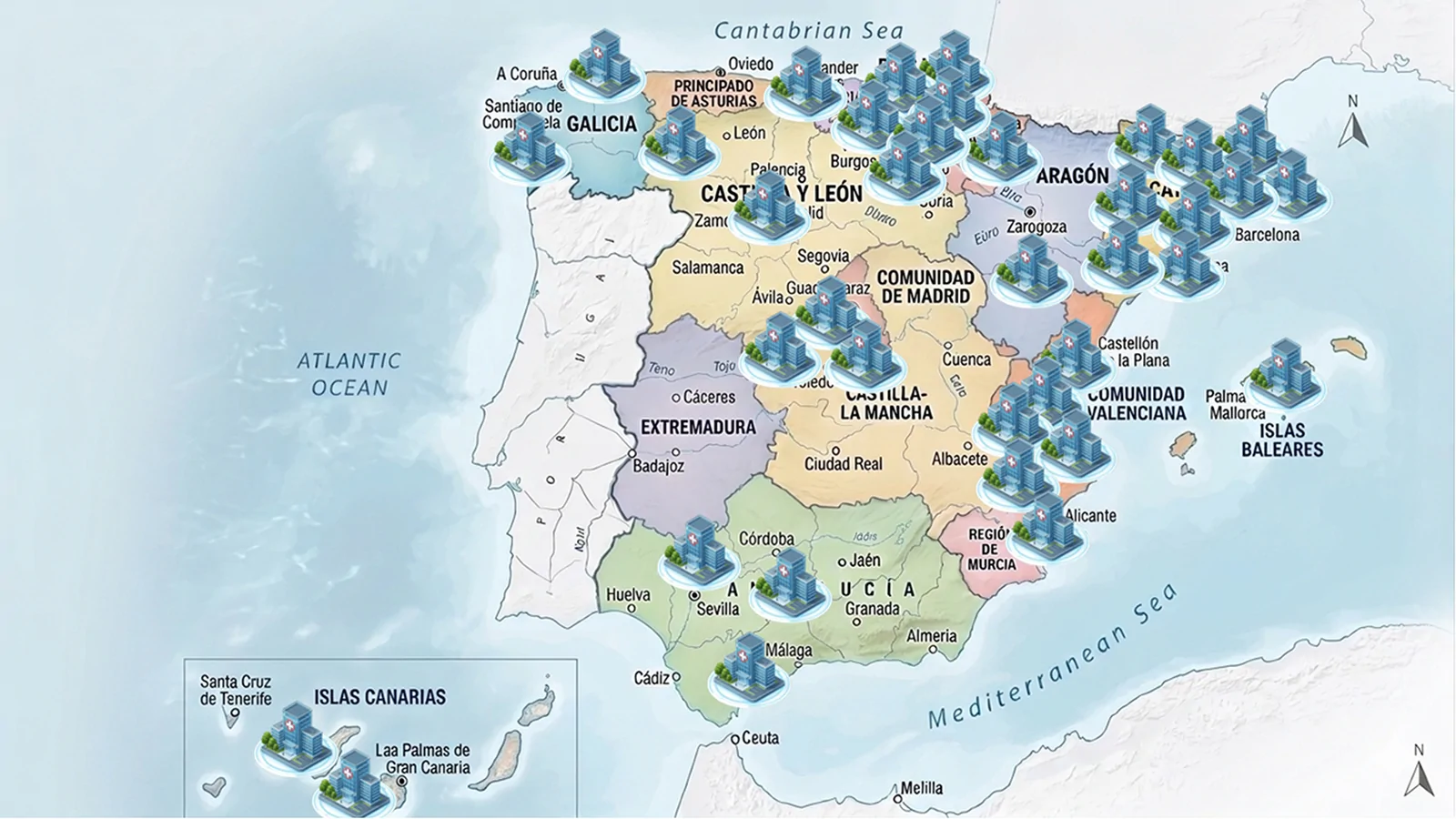
Participating hospitals across Spain

61 VASTs were contacted, of which 37 (60.65%) agreed to participate (see Fig. 1). Finally, 51 VAST nurses in Spain completed the questionnaire. The sample was predominantly female (72.5%), with a mean age of 44.5 years (SD ± 9.5). Participants had 21 years of professional experience (SD ± 9.7), and 3.8 years (SD ± 3.6) of involvement in their respective VASTs.

The overall mean score across the seven dimensions of the questionnaire was 4.2 out of 5. The highest-rated domain was Social/Professional role and identity (Domain 3), with a mean of 4.7 (SD ± 0.5). This was followed by Knowledge (D1) and Collective context (D6), both with mean scores above 4.2. Conversely, the Context (D5) dimension received the lowest score (mean 3.9, SD ± 0.5). All data are included in Table 1.

**Table 1:** Sociodemographic characteristics and questionnaire scores

Characteristics	*N* (%)	Mean (SD)	Minimum	Maximum	Range
Gender	51 (100)				
Male	13 (25.5)				
Female	37 (72.5)				
Nonbinary	1 (2.0)				
Age	51 (100)	44.47 (9.52)	24	61	37
Years of experience	51 (100)	21 (9.67)	1	40	39
Years of VAST experience	51 (100)	3.75 (3.63)	0	15	15
**Dimensions** of the questionnaire					
D1 Knowledge	51 (100)	4.298 (0.44)	3.20	5.00	1.80
D2 Skills	51 (100)	4.196 (0.46)	3.00	5.00	2.00
D3 Social/professional role and identity	51 (100)	4.657 (0.48)	3.50	5.00	1.50
D4 Beliefs about capabilities	51 (100)	4.049 (0.47)	3.20	5.00	1.80
D5 Context	51 (100)	3.922 (0.50)	3.00	5.00	2.00
D6 Collective context	51 (100)	4.186 (0.55)	3.00	5.00	2.00
D7 Behavioural regulation	51 (100)	4.137 (0.72)	3.00	5.00	2.00

Abbreviations: SD, standard deviation; VAST, vascular access specialist team; D1–D7, questionnaire domains (D1, Knowledge; D2, Skills; D3, Social/professional role and identity; D4, Beliefs about capabilities; D5, Context; D6, Collective context; D7, Behavioural regulation)

The scores obtained regarding agreement among participants (Table 2) demonstrated a strong sense of professional responsibility and role clarity regarding the implementation of intravenous therapy improvement interventions. Over 90% of participants reported high levels of knowledge, clarity about their responsibilities, and confidence in their skills to implement these interventions. Notably, 100% (51/51) recognised it as part of their professional role. However, lower agreement was observed in items related to perceived behavioural control, with only 58.8% (30/51) stating that implementation was “very easy” and around 70% (36/51) feeling in full control of the process.

**Table 2:** Scores for each questionnaire item regarding agreement among participants (Likert scale values 4–5)

Domain	Constructs (Number items)	Value 4–5 (%)
D1 Knowledge	Knowledge (1)	
	D1.1 I know how to implement intravenous therapy improvement interventions.	96.1
	Role clarity (4)	
	D1.2 The objectives of my role and the interventions I need to implement are clearly defined for me.	94.1
	D1.3 I know what my responsibilities are regarding the implementation of intravenous therapy improvement interventions.	92.2
	D1.4 Regarding the implementation of IVT improvement interventions, I know exactly what is expected of me (my supervisor and/or manager).	80.4
	D1.5 The interventions to be carried out are clearly defined for me.	94.1
D2 Skills	Skills (3)	
	D2.1 I have received training in implementing intravenous therapy improvement interventions.	88.2
	D2.2 I have the necessary skills to implement intravenous therapy improvement interventions.	90.2
	D2.3 I can implement intravenous therapy improvement interventions.	90.2
D3 Social/professional role and identity	Professional role (2)	
	D3.1 Implementing intravenous therapy improvement interventions is part of my job as a nurse of vascular access specialist team.	96.1
	D3.2 It is my responsibility as a nurse in the VAST to implement intravenous therapy improvement interventions.	100
D4 Beliefs about capabilities	Self-efficacy (4)	
	D4.1 I am confident in being able to implement intravenous therapy improvement interventions.	94.1
	D4.2 I am confident in being able to implement intravenous therapy improvement interventions even when other professionals do not.	92.2
	D4.3 I am confident in being able to implement intravenous therapy improvement interventions even when there is time available to do so.	90.2
	D4.4 I am confident in being able to implement intravenous therapy improvement interventions even when the targeted professionals are not motivated.	80.4
	Perceived behavioural control (6)	
	D4.5 I am in control of how the implementation of intravenous therapy improvement interventions will be carried out.	72.5
	D4.6 Implementing intravenous therapy improvement interventions is very easy for me.	58.8
	D4.7 Delivering training related to an intravenous therapy improvement intervention is very easy for me.	80.4
	D4.8 Evaluating participants who receive training related to an intravenous therapy improvement intervention is very easy for me.	80.4
	D4.9 Monitoring the maintenance of optimal intravenous therapy maintenance behaviours outside of an intravenous therapy improvement intervention is very easy for me.	76.5
	D4.10 Providing feedback on the intravenous therapy improvement intervention to the professional targeted by the intervention is easy for me.	70.6
D5 Context	Social-political context (1)	
	D5.1 The team receives sufficient support from the government and local authorities for the implementation of interventions to improve intravenous therapy.	72.5
	Organisational resources and support (4)	
	D5.2 In the organisation where I work, I have all the necessary resources to implement intravenous therapy improvement interventions.	66.7
	D5.3 I have the support of the management of the organisation in which I work for the implementation of intravenous therapy improvement interventions.	68.6
	D5.4 The management of the organisation is open to listen to the difficulties in implementing intravenous therapy improvement interventions.	60.8
	D5.5 The management of the organisation in which I work offers support (financial, resources, training, etc.) for the implementation of intravenous therapy improvement interventions.	72.5
	Innovation strategy (1)	
	D5.6 The organisation in which I work provides training for professionals in the implementation of intravenous therapy improvement interventions.	74.5
D6 Collective context	Social influences (2)	
	D6.1 The professionals with whom I implement intravenous therapy improvement interventions think that I should be the one to implement and/or lead them.	72.5
	D6.2 The existence of vascular access specialist team professionals from other centres carrying out implementation has an influence on me.	92.2
D7 Behavioural regulation	Action planning (1)	
	D7.1 I have a clear plan for how I would deliver an intravenous therapy improvement intervention.	80.4

Correlation analyses revealed significant positive relationships between key domains: D1 (Knowledge) and D4 (Beliefs about Capabilities): ρ = 0.574, *p* < 0.01; D1 and D7 (Behavioural Regulation): ρ = 0.591, *p* < 0.01; D2 (Skills) and D4: ρ = 0.548, *p* < 0.01; and D4 and D6 (Organisational Context): ρ = 0.509, *p* < 0.01. All data are included in Table 3.

**Table 3:** Correlations between domains related to implementation questionnaire

Variables	D1	D2	D3	D4	D5	D6	D7
**D1**, ρ (p-value)	1	0.454 (0.001)	0.192 (0.178)	0.574 (< 0.001)	0.296 (0.035)	0.300 (0.032)	0.591 (< 0.001)
**D2**, ρ (p-value)		1	0.156 (0.275)	0.548 (< 0.001)	0.329 (0.018)	0.288 (0.040)	0.364 (0.009)
**D3**, ρ (p-value)			1	0.309 (0.309)	0.114 (0.424)	0.372 (0.007)	0.160 (0.262)
**D4**, ρ (p-value)				1	0.258 (0.068)	0.509 (< 0.001)	0.332 (0.017)
**D5**, ρ (p-value)					1	0.279 (0.047)	0.143 (0.246)
**D6**, ρ (p-value)						1	0.246 (0.082)
**D7**, ρ (p-value)							1

D1. Knowledge; D2. Skills; D3. Social/professional role and identity; D4. Beliefs about capabilities; D5. Context; D6. Collective context; and D7. Behavioural regulation)

No statistically significant differences were found between groups based on age, years of professional experience, or time working in a VAST, across the domains of the implementation questionnaire. However, a statistically significant difference was observed between years of experience and the collective context domain. Detailed results are presented in Table 4.

**Table 4:** Relationship of sociodemographic characteristics and the domains of the implementation questionnaire

Variables	D1	D2	D3	D4	D5	D6	D7
**Age**, ρ (p-value)	0.246 (0.082)	0.20 (0.891)	0.213 (0.133)	0.037 (0.797)	0.068 (0.637)	0.108 (0.452)	0.232 (0.101)
**Gender**, ρ (p-value)	0.493 (0.486)	0.245 (0.623)	0.798 (0.376)	2.399 (0.128)	1.572 (0.216)	0.532 (0.469)	0.001 (0.971)
**Years of experience**, ρ (p-value)	0.198 (0.163)	-0.048 (0.737)	0.235 (0.097)	0.087 (0.543)	-0.037 (0.797)	0.117 (0.413)	0.215 (0.130)
**Years of VAST experience**, ρ (p-value)	0.173 (0.225)	0.078 (0.588)	0.264 (0.061)	0.102 (0.476)	0.039 (0.784)	0.293 (0.037)	0.208 (0.143)

### Qualitative stage

The qualitative stage included interviews with ten nurses from VASTs in diverse hospital contexts. Thematic analysis revealed five overarching themes that collectively portrayed how VAST nurses enacted and developed implementation competencies in the absence of formal training specific to implementation, despite receiving routine technical and device-related instruction. Appendix 3 provides a matrix of themes, subthemes, and illustrative quotes.

#### Expanded professional role

Participants highlighted a transformation in their role, from a primarily technical focus on vascular access device insertion to a broader scope that included education, consultancy, auditing, epidemiological monitoring, and leadership in quality improvement. While these activities extended beyond clinical expertise, they also reflected the early stages of an implementation-oriented role, as VAST nurses increasingly influenced how others delivered care, guided decision-making, and embedded best practices into routine workflows. Participants widely agreed that VASTs should evolve beyond a technical insertion team to become a reference service for driving practice change, supporting not only device selection but also the adoption and reinforcement of evidence-based behaviours across clinical units.

This expanded role was closely mediated by organisational context. In smaller hospitals, greater autonomy and flatter hierarchies enabled VAST nurses to influence ward routines more directly and to embed informal mechanisms that supported consistent and evidence-based vascular access care. These contextual features provided greater flexibility to extend their scope and to accelerate the implementation of practice change.

*The VAST team is not just about inserting lines; there’s a lot of work behind the scenes.* (P1)

*Every day is different. I do insertion*,* follow-up*,* answer consultations*,* prepare teaching materials… we do everything.* (P10)

The daily responsibilities of VAST nurses extended beyond device insertion to include ongoing clinical assessments, staff education, and consultative support. Participants described providing bedside guidance, modelling best practices and tailoring training to ward needs, a set of activities that actively shaped the behaviour of other clinicians. This multidimensional role positioned them not only as clinical referents but also facilitators of practice change, helping teams adopt and sustain safer vascular access procedures.

*We give training on the ward, advise on which device to use, and solve problems when there are complications*. (P2)

#### Implementation competencies

None of the participants had received formal education in implementation science. Learning occurred primarily through experiential processes, including trial-and-error, mentorship, and peer observation. Despite this lack of structured training, participants described a range of behaviours and skills that aligned with core implementation competencies, such as planning change initiatives, adapting interventions to local context, troubleshooting barriers, engaging stakeholders and monitoring outcomes over time.

*We do it intuitively… I guess it’s a science of some sort.* (P1)

*What I used to hate—planning*,* metrics*,* follow-up—I now realise are essential.* (P9)

Across interviews, participants demonstrated elements of implementation capability, even if not framed as such. Many described systematically identifying problems, developing pragmatic solutions, testing and refining strategies, and using local data to inform decisions – processes that mirrored iterative cycles of improvement and facilitation found in implementation frameworks such as i-PARIHS and COM-B. These behaviours suggested an emerging but largely untapped competence that could be strengthened through formal development. Participants also expressed a strong desire for structured training to consolidate their intuitive practices and to build confidence in applying evidence-informed methods. They perceived that formal education would enable them not only to improve their own performance but also to lead change more effectively, engage colleagues, and sustain change within their organisations.

*If there was a course on this*,* I’d do it without hesitation. It would help us a lot.* (P4)

*I’m interested in knowing how to make changes stick over time.* (P7)

#### Implementation strategies

VAST nurses employed a wide range of context-responsive implementation strategies to promote behaviour change and improve adherence to best practices. Training was typically delivered on-site, at the bedside or during shift transitions, ensuring immediate relevance and minimising disruption to routine workflows. Materials included short videos, infographics, posters, and digital messages, which were used to reinforce key practices in accessible formats. These approaches were consistent with well-established strategies, such as conducting educational meetings, using educational materials and adapting interventions to local context.

*When we detect a problem on a ward*,* we go there and deliver a brief session on the spot.* (P3)

*We track all insertions and complications ourselves. That data helps justify changes.* (P10)

*We’ve made short videos and digital infographics*,* anything that helps people remember.* (P8)

Participants also described extensive use of local data systems, often created and maintained independently of formal quality structures. They collected and analysed data on complication rates, device outcomes and ward-level trends to guide decision-making and support targeted interventions. This data-driven approach enabled iterative cycles of assessment, adaptation, and re-evaluation, mirroring the logic of quality improvement and implementation frameworks.

*We analyse phlebitis rates by ward. If a unit shows high rates*,* we act. *(P1)

*Every month we review data and discuss it with supervisors.* (P2)

By combining real-time observation, customised education and continuous monitoring, VAST nurses applied practical facilitation strategies that enhanced their credibility, supported behaviour change and increased the likelihood that improvements would be sustained over time, even in the absence of formal organisational support structures.

#### Organisational barriers

All participants identified structural limitations as a key challenge. Most professionals reported insufficient staffing and the absence of dedicated time for implementation activities such as staff training, data analysis, or process evaluation. Their implementation efforts often occurred on top of routine care, leading to overload and reactivity rather than proactive planning.

*We do everything when we are already overwhelmed. There’s no protected time. *(P5)

*We lack time.* (P6)

Despite the importance and visibility of their work, many participants noted that their role was not formally recognised in hospital organisational charts or strategic plans. This lack of formalisation undermined the sustainability and legitimacy of the team’s efforts and made it harder to advocate for additional resources or integration in decision-making bodies.

*We’re seen as important*,* but not yet structured into the organisation.* (P2)

*Management trusts us*,* but it’s not formalised. There’s no structural role.* (P5)

Frequent changes in hospital staff, especially in wards, were cited as a major barrier. Implementation efforts—such as training or behavioural reinforcement—often had to be restarted from scratch, leading to fatigue and a sense of stagnation among VAST nurses.

*Staff changes so often that by the time you finish training some*,* you have to start again with others*. (P7).

*Lots of temporary staff*,* and that’s noticeable*. (P10)

At the same time, many of the organisational barriers —including limited time, workforce instability and the absence of formalised structures —mirrored challenges commonly reported across clinical settings. However, these conditions had particular consequences for VAST teams, whose implementation work depends on continuity, organisational support and consistent engagement with ward staff. Consequently, such systemic constraints may have disproportionately hindered their ability to initiate, scale or maintain practice change, creating an environment with limited capacity to support VAST-led implementation efforts despite the motivation and expertise of these professionals.

#### Enabling local conditions

When implementation efforts were successful, participants cited strong support from nursing leadership, informal recognition by colleagues, and collaboration across departments. They also described an organisational climate that supported professional autonomy and communication within teams. Involvement in multidisciplinary committees and the use of internal data were additionally viewed as legitimising factors that strengthened their authority to influence practice.

*The nursing management supports us*,* and that makes all the difference.* (P3)

*We spotted a problem and channelled it through our Best Practice Spotlight Organisation programme lead. That connection is key.* (P10)

In addition to these existing facilitators, participants articulated several desired conditions that they believed would further enhance their capacity to implement and sustain change. These included the formal recognition of the VAST role, as well as partial or full-time role protection to ensure structured and continuous training, dedicated improvement activities and greater strategic involvement. They also emphasised the need to develop inter-institutional networks, enabling VAST teams across centres to share experiences, harmonise approaches and collectively strengthen vascular access practices at a national level.

*We need at least two more nurses and a second VAST at the other hospital. (P7)*

*We should have meetings between VASTs from different centres*,* we would learn a lot. (P1)*

Together, these conditions illustrated how organisational support, professional autonomy and networked collaboration could create a more favourable implementation environment.

### Mixed-methods integration

To synthesise the quantitative and qualitative findings, a mixed-methods joint display was constructed (Table 5). This matrix illustrated how survey domains aligned with qualitative themes and subthemes, enabling the generation of integrated meta-inferences regarding VAST implementation competence.

**Table 5:** Joint display of integrated mixed-methods findings

Quantitative analysis		Qualitative analysis		Integrated interpretation
Domain	Findings	Themes	Subthemes / Codes	
D1. Knowledge	96.1% report knowing how to implement interventions	Implementation competencies	Empirical and informal acquisition	**High convergence**: professionals feel knowledgeable, and interviews confirm deep experiential knowledge—even without formal training. Knowledge is acquired informally and pragmatically.
	90% report knowing objectives, responsibilities and expectations			
D2. Skills	High perceived skills (88–90%). Most feel capable of implementing interventions		Desire for formal training	**Partial convergence**: perceived skills are high, but interviews reveal skills learned informally and a lack of formal education. Gap between perceived vs. formal competency.
	88.2% say they have received “training”.			**Key divergence**: wording of the item likely misinterpreted → important finding demonstrating the need for formal implementation training.
D3. Professional role and identity	Highest scoring domain (M = 4.7). 96–100% agree implementation is part of their role. Strong role clarity.	Expanded professional role	From technical insertion to integral leadership	**Strong convergence**. Both datasets show consolidated identity as implementers.
			Clinical, formative and consultative functions	Nurses clearly perceive implementation as part of their job and describe actions aligned with this identity.
D4. Beliefs about capabilities	High self-efficacy but lower perceived behavioural control (only 58.8% find implementation “easy”).	Organisational barriers	Lack of human resources and protected time	**Strong triangulation**: self-confidence is high, but organisational barriers reduce perceived ease, aligning low D4.6 values with narratives of overload and structural gaps.
D5. Context (social–political organisational)	Lowest scoring domain. Only 60–70% feel organisational support; <61% feel leadership listens.		Lack of structural recognition	**Full convergence**: interviews strongly emphasise insufficient structural support, matching low D5 values. Organisational factors = primary barrier.
			High staff turnover	
			Lack of resources and time	
D6. Social influences / collective context	High influence from peers and other VASTs (92.2% report positive impact).	Enabling conditions	Collaborative culture and networking	**Total convergence**: collaboration, peer influence, and leadership support emerge as key facilitators in both datasets.
			Support from leadership and professional recognition	
D7. Behavioural regulation	Good planning (80.4%). Strong correlations D1–D7 (ρ = 0.59) and D2–D4 (ρ = 0.54).	Implementation strategies	On-site, tailored, context-sensitive training	**Strong convergence**: qualitative strategies align precisely with behavioural regulation (planning, adapting, monitoring). Data-driven decisions match strong D1–D7 correlation.
			Visual and informal communication	
			Use of data and iterative improvement	

The integration revealed areas of convergence, such as the alignment between high self-perceived knowledge, strong professional identity and the qualitative descriptions of adaptive, context-sensitive strategies used in daily practice. It also demonstrated complementarity, with the qualitative data expanding survey findings by detailing how intuitive, experiential learning compensates for the absence of formal implementation training. This integration highlighted divergences, particularly between the relatively positive quantitative ratings of organisational support and the qualitative accounts of structural limitations, insufficient recognition and lack of protected time. These integrated insights provided a more comprehensive understanding of the mechanisms, contextual influences and gaps between capability and context that shape VAST nurses’ implementation competence.

## Discussion

This study explored implementation competence for EBP among vascular access specialist nurses in Spain using a mixed-method approach. Combining quantitative and qualitative data provided a comprehensive understanding of how these nurses perceive, develop and enact their role in translating evidence into routine clinical practice. The findings revealed both convergence and divergence between the responses of professionals to structured scales and their descriptions of daily practice, offering nuanced insights into the strengths and weaknesses of implementation in real-world settings. Together, these findings help explain how individual-level competencies intersect with contextual and organisational determinants to enable or constrain implementation, in line with established implementation frameworks that emphasise the dynamic interaction between individuals, context and facilitation processes [35, 36].

The results showed a high level of agreement on the clarity of roles, professional identity and confidence in knowledge and skills. These findings were corroborated in the qualitative interviews, where participants demonstrated a clear understanding of their responsibilities and outlined various strategies employed in daily practice to improve intravenous therapy. A strong sense of ownership of the implementation role emerged, reflecting a shared professional commitment to influencing practice. This alignment between perceived competence and observed practice suggests that self-efficacy is a central driver of nurses’ engagement with EBP, consistent with evidence identifying self-efficacy as a key determinant of EBP adoption and implementation behaviour among healthcare professionals [37].

Despite lacking formal instruction in implementation science, participants described strategies that aligned with key implementation principles, such as tailored communication, local data use and in-situ training, but the qualitative data highlighted important structural and contextual limitations. The perceived behavioural control domain (D4) showed significantly lower agreement on items assessing ease of implementation and control. This was consistent with narratives about barriers to engaging in EBP and practice improvement activities, which emphasised a lack of time, staffing shortages, and lack of structural recognition [38, 39]. Such conditions are well-documented barriers that limit the feasibility of EBP in routine care.

Although this study was not explicitly designed using a predefined implementation framework, many of the processes described by participants reflect key components of established models such as COM-B and i-PARIHS [35, 40]. In particular, participants described identifying local barriers and facilitators, adapting interventions to context, and using data to guide iterative improvement. These findings suggest that VAST nurses are enacting implementation practices that are theoretically consistent, despite the absence of formal training or explicit use of implementation frameworks.

Tensions emerged regarding training, with participants reporting that most educational opportunities focused on technical or device-related aspects rather than on skills required to implement and sustain EBP. This finding aligns with previous studies showing that proficiency in clinical procedures does not necessarily translate into competence in leading or supporting EBP-related change [41]. Notably, although a majority of participants reported some degree of organisational support, qualitative findings revealed limited institutional visibility of the VAST role with broader EBP and quality strategies. Participants described reliance on individual goodwill rather than structured organisational facilitation, suggesting that EBP in nursing is frequently maintained through personal commitment in the absence of consistent system-level support. These findings are consistent with international evidence that EBP adoption often stalls when nursing roles are not embedded within supportive organisational and policy frameworks [19, 28, 42]. Such organisational challenges also reflect broader system-level constraints (CFIR outer setting), including limited institutional recognition of implementation roles within national healthcare frameworks and the absence of standardised pathways for integrating evidence into policy and practice [36].

Integrating both datasets suggests that highly motivated nurses with strong EBP capability are operating within constrained organisational environments. This interaction between self-efficacy, behavioural regulation, and contextual support aligns with established behavioural and implementation perspectives, which emphasise that sustained EBP requires both individual capability and organisational readiness [37]. Although vascular access specialist nurses appear to possess strong foundational skills and intrinsic motivation to lead EBP, their capacity to do so is constrained by inadequate structural and organisational support. This finding reflects a well-documented challenge in the EBP and implementation literature, namely the misalignment between individual readiness for change and organisational readiness to support and sustain practice change [36].

Several hypotheses may explain the observed lack of formal training in implementation methodologies among VAST professionals. One possible reason is the relative novelty of the VAST role or model within our healthcare context. Being a recently established professional group, formalised training programs and educational pathways focused on implementation competencies might still be under development or limited in availability. This lack of formalised training and inconsistent organisational recognition suggests an absence of shared professional consensus regarding the implementation-related responsibilities that VAST members are expected to assume. Establishing such consensus is therefore a necessary preliminary step before competencies can be formally developed, taught, and integrated into professional practice. This reflects broader challenges described in implementation scholarship, where professional groups often acquire implementation roles before structured training pathways are established [43]. Finally, organisational factors such as limited resources, competing priorities, or insufficient institutional support may also contribute to this gap [38, 39, 41].

Understanding and addressing these underlying causes is essential for designing effective strategies to build implementation capacity in VASTs. To enhance the implementation capacity of VASTs, a dual strategy is required. Firstly, there should be sustained investment in formal education, and secondly, mentoring in implementation frameworks must be provided by organisational programmes, alongside the creation of supportive institutional conditions. This encompasses formally recognising the VAST role as implementers, allocating time for conducting interventions, and incorporating VASTs into strategic quality committees. Implementing these measures could enhance the effectiveness of improvement interventions and ultimately contribute to the broader objective of embedding evidence-based practices in routine clinical work [44].

This study should be interpreted with consideration of several limitations. The absence of an official national registry of VASTs in Spain meant that participants from different geographical regions could not be identified and recruited systematically. This may have resulted in unbalanced geographical representation and sampling bias, potentially limiting the external validity of the findings. Moreover, because recruitment relied predominantly on large, tertiary, and university-affiliated hospitals, which often have more mature VAST structures and stronger organisational infrastructures, the findings may overestimate the level of implementation competence or organisational support available in smaller or less resourced institutions. This limits the transferability of the results to contexts where VAST teams are newer, less formalised, or operate with fewer institutional enablers. Furthermore, using a Likert-scale questionnaire to explore constructs with which many participants may be unfamiliar –particularly those lacking formal training in implementation science– introduces the possibility of response bias. This bias may manifest as socially desirable answers [45] or a disproportionate response rate from nurses who are already engaged with, motivated by, or sensitised to implementation-related issues. This is consistent with the lower levels of agreement observed in the perceived behavioural control domain, where unfamiliarity with implementation concepts may have influenced how participants approached the items. Additionally, self-reported data may be unreliable due to differences in how individuals interpret items and variations in respondents’ understanding of the theoretical constructs being measured. Nevertheless, the inclusion of qualitative data provided substantial contextual depth, allowing cross-validation of quantitative patterns and enhancing interpretive rigour. Taken together, this mixed-method triangulation helped mitigate some of these limitations and strengthened the credibility and interpretive validity of the findings. These methodological constraints suggest that future efforts to strengthen VAST implementation competence should move beyond reliance on individual motivation and towards approaches that embed implementation capability as an organisational function.

## Conclusion

This study provides the first in-depth characterisation of implementation competence for EBP among VAST nurses in Spain. Using a mixed-method approach, we identified a highly motivated and experienced workforce that views EBP as integral to its professional role, supported by strong role clarity, knowledge, and adaptive practice strategies. Despite these strengths, a substantial gap emerged between individual readiness for EBP and organisational capacity to support, which restricted nurses’ perceived control over practice change. These findings highlight the need to strengthen both individual capability and contextual enablers to ensure that VASTs can function as effective leaders of EBP.

A dual strategy is therefore required, combining targeted education aligned with EBP and implementation frameworks with organisational conditions that formally recognise and integrate VAST roles within quality and patient safety strategies. At a system level, national mechanisms that enhance coordination, role visibility, and standardisation may further support the sustainability and scale-up of evidence-based vascular access practices across healthcare settings.

## Electronic supplementary material

Below is the link to the electronic supplementary material.

Supplementary Material 1

## Data Availability

The datasets generated during and/or analysed during the current study are/will be available upon request from the corresponding author on reasonable request and will be included in the published article (and its supplementary information files). The data will be available beginning 3 months and ending 5 years following article publication for to investigators, whose proposed use of data will have been approved by an independent review committee identified for to realise the qualitative meta-analysis. Proposals may be submitted up to 36 months following article publication.

## Abbreviations

VAD: Vascular access device
VAST: Vascular access specialist team
EBP: Evidence-based practice
CRBSI: Catheter-related bloodstream infection
COM-B: Capability, Opportunity, Motivation-Behaviour model
i-PARIHS: Integrated Promoting Action on Research Implementation in Health Services framework
