# Supplementary material for: Implementation competence for evidence-based practice among vascular access specialist team nurses: a mixed-method study
Source: BMC Nurs. 2026 Jun 17;25:911. doi: 10.1186/s12912-026-04783-w (PMC13629114; doi:10.1186/s12912-026-04783-w)
Supplement: Supplementary file 1 [file 12912_2026_4783_MOESM1_ESM.docx]

IMPLEMENTATION COMPETENCE FOR EVIDENCE-BASED PRACTICE AMONG VASCULAR ACCESS SPECIALIST NURSES: A MIXED-METHOD STUDY by Ismael Fernández-Fernández et al.

**Appendix 1. Domain definitions of final questionnaire for measuring of implementation behaviour.**

| **Domains** | | **Definition** |
| --- | --- | --- |
| D1 | Knowledge | An awareness of the existence of something. |
| D2 | Skills | An ability or proficiency acquired through practice. |
| D3 | Social/professional and role and identity | A coherent set of behaviours and displayed personal qualities of an individual in a social or work setting. |
| D4 | Beliefs about capabilities | Acceptance of the truth, reality, or validity about an ability, talent, or facility that a person can put to constructive use. |
| D5 | Socio-political context | -Any characteristics of the socio-political context that discourages or encourages the development of skills and abilities, independence, social competence, and adaptive behaviour.  -Any characteristics of the organization that discourages or encourages the development of skills and abilities, independence, social competence, and adaptive behaviour.  -Any characteristics of the innovation strategy that discourages or encourages the development of skills and abilities, independence, social competence, and adaptive behaviour. |
| D6 | Collective context | Those interpersonal processes that can cause individuals to change their thoughts, feelings, or behaviours. |
| D7 | Behavioural regulation | Anything aimed at managing or changing objectively observed or measured actions. |

**Appendix 2. Script for the semi-structured interviews**

1. Tell us briefly about your typical day as a VAST nurse.
   a. *Probe: What kind of activities he/she carries out, with whom he/she interacts, etc.*
2. Considering the activities that you have just mentioned, how would you describe your role should any of these activities require improving?
   a. *Probe: E.g., high adverse effect rates, etc.*
   b. *Probe: What type of interventions would he/she carry out.*
3. *[If the interviewee plays a role in improvement interventions]* How do you carry out the quality improvement interventions that relate to intravenous therapy and vascular access care? *[OR]* How are the interventions carried out?
   a. *Probe: Explore whether the interventions are planned processes based on some type of model or evidence.*
   b. *Probe: Explore whether the interventions involve the ‘affected’ unit, team, or patient.*
   c. *Probe: In case they provide any evidence, explore what their formal training is, the training they have received, what practical experience they have from previous similar interventions, etc.*
4. Within the context of your unit, what factors would you say influence (facilitate or hinder) the implementation of interventions aimed at improving intravenous therapy and vascular access?
   a. *Probe: What do your peers, other nurses, think about what you should do or know in this area?*
   b. *Probe: What do you think your peers, other nurses, should do or know in this area?*
5. *[Ask the interviewee about implementation assessment]*. What kind of impact do you think implementation skills have on your unit and on you (no impact, a negative impact, a positive impact)?
6. What aspects of the development/implementation of improvement interventions could be improved?
7. What is the first thing that comes to your mind when you hear someone say ‘implementation’ and ‘implementation skills’?
   a. *Probe:* *Reflecting on everything we have said, the entire process, the whole experience, etc.*
8. Is there anything you would like to add that we haven’t covered in this interview?

**Appendix 3. Themes and sub-themes identified on implementation competence in VAST nurses**

| **Themes** | **Subthemes** | **Verbatim** |
| --- | --- | --- |
| Expanded Professional Role | From technical insertion to integral leadership | “*The VAST team is not just about inserting lines; there’s a lot of work behind the scenes.”* (P1)  “*Every day is different. I do insertion, follow-up, answer consultations, prepare teaching materials... we do everything.*” (P10) |
|  | Clinical, formative, and consultative functions | “We give training on the ward, advise on which device to use, and solve problems when there are complications.” (P2)  “We have a follow-up agenda to check catheter performance.” (P8) |
| Implementation competencies | Empirical and informal acquisition | “*No formal training, just what I’ve learned from experience and observation.”* (P10)  “*We do it intuitively… I guess it’s a science of some sort.*” (P1)  “*What I used to hate—planning, metrics, follow-up—I now realise are essential.*” (P9) |
|  | Desire for formal training | “*If there was a course on this, I’d do it without hesitation. It would help us a lot.*” (P4)  “*I'm interested in knowing how to make changes stick over time.”* (P7) |
| Implementation strategies | On-site, tailored, context-sensitive training | “*After a spike in phlebitis cases, we’re planning a session with doctors to address it.*” (P10)  “*When we detect a problem on a ward, we go there and deliver a brief session on the spot.*” (P3)  “*We track all insertions and complications ourselves. That data helps justify changes.*” (P10) |
|  | Visual and informal communication | “*We’ve made short videos that play on ward computers; they work very well*.” (P8)  “*We’ve made short videos and digital infographics, anything that helps people remember.*” (P8)  “*We even created mobile-based games like Kahoot to reinforce learning.”* (P6)  “*Posters, reminders in restrooms—anything eye-catching*.” (P7) |
|  | Use of data and iterative improvement | “*We analyse phlebitis rates by ward. If a unit shows high rates, we act*.” (P1)  “*We log all devices in our own database*.” (P10)  “*Every month we review data and discuss it with supervisors*.” (P2) |
| Organisational barriers | Lack of human resources and protected time | “*We don’t have dedicated staff for this*.” (P5)  “*We lack time*.” (P6)  “*We improvise based on whatever comes up during the day*.” (P5) |
|  | Lack of structural recognition | “*Management trusts us, but it’s not formalised. There’s no structural role*.” (P5)  “*We do a lot, but we’re still seen as an add-on, not a consolidated team*.” (P2) |
|  | High staff turnover | “*Staff changes so often that by the time you finish training some, you have to start again with others*.” (P7)  “*Lots of temporary staff, and that’s noticeable*.” (P10) |
| Enabling local conditions | Support from leadership and professional recognition | “*Nursing leadership supports us, and that makes all the difference*.” (P3)  “*Our results speak for themselves: fewer complications, better device use*.” (P4)  “*This team exists because management wanted it to*.” (P10) |
|  | Collaborative culture and networking | “*We should have meetings with VASTs from other centres; we’d learn a lot*.” (P9)  “*We identified the problem and routed it through the BPSO lead*.” (P10)  “*We’re in constant communication with doctors and supervisors*.” (P6) |
